# Can Endoscopic Appearance, Selective Cytology, and Pathological Sampling During Ureteroscopy Accurately Predict Tumor Grade of Upper-Tract Urothelial Carcinoma?

**DOI:** 10.5041/RMMJ.10459

**Published:** 2022-01-27

**Authors:** Kamil Malshy, Omri Nativ, Ariel Zisman, Omer Sadeh, Azik Hoffman, Gilad E. Amiel, Michael Mullerad

**Affiliations:** Department of Urology, Rambam Health Care Campus, Haifa, Israel

**Keywords:** Diagnostic ureteroscopy, endoscopic appearance, endoscopic biopsy, tumor grade, upper-tract urothelial carcinoma, urine cytology, UTUC

## Abstract

**Objective:**

This study examined the reliability of the various parameters obtained in diagnostic ureteroscopy for upper-tract urothelial carcinoma (UTUC) in predicting the degree of differentiation in the final pathological report after radical nephroureterectomy (RNU).

**Methods:**

We conducted a retrospective review of patients undergoing RNU at a single tertiary hospital between 2000 and 2020. Only patients who underwent preoperative diagnostic ureteroscopy (URS) were included. The results of urine selective cytology, endoscopic appearance of the tumor, and biopsy taken during ureteroscopy were compared to the final pathological report.

**Results:**

In total, 111 patients underwent RNU. A preliminary URS was performed in 54. According to endoscopic appearance, 40% of the “solid”-looking tumors were high grade (HG), while 52% of those with a papillary appearance were low grade (LG). Positive cytology predicted HG tumors in 86% of cases. However, 42% of patients with negative cytology had HG disease. The biopsies acquired during URS showed that HG disease findings matched the final pathology in 75% of cases. However, 25% of patients noted as being HG, based on URS biopsies, were noted to have LG disease based on nephroureterectomy biopsies. Full analyses revealed that 40% of the cases diagnosed as LG based on the URS biopsies actually had HG disease.

**Conclusions:**

Direct tumor observation of papillary lesions, negative cytology, and biopsies indicating LG disease are of low predictive value for classifying the actual degree of tumor differentiation. No single test can accurately rule out HG disease. In light of the rising use of neo-adjuvant chemotherapy in UTUC, a reliable predictive model should be developed that accurately discriminates between HG and LG disease.

## INTRODUCTION

Upper-tract urothelial carcinoma (UTUC) accounts for 5%–10% of urothelial carcinomas (UC),^1^ with an annual incidence of 2:100,000 in Western countries.^2^ Males have a three-fold increased risk compared to women.^3^ The most known risk factors are cigarette smoking, arsenic exposure, Balkan nephropathy, and hereditary non-polyposis colorectal cancer.^4^

While in the past radical nephroureterectomy (RNU) with bladder cuff excision was overwhelmingly the treatment of choice, other less invasive treatments were used only for exceptional cases such as a single functioning kidney, bilateral disease, and distal ureteral focal disease; however, in recent years patient-specific strategies have emerged.^5^

The updated European Urological Association guidelines of 2021 recommend renal sparing surgeries for low-risk disease (unifocal, <2 cm, low-grade [LG] cytology, LG biopsy, and not invasive on computed tomography [CT]), most commonly by ureteroscopic resection or distal ureterectomy, allowing for ipsilateral kidney preservation without oncological compromise. High-risk disease (hydronephrosis, >2 cm, high-grade [HG] cytology, HG biopsy, multifocal, or previous radical cystectomy for bladder urothelial carcinoma and variant histology) is still treated with RNU as the gold standard, although there is an increasing use of neoadjuvant platin-based chemotherapy.^6^

Given the above, diagnostic ureteroscopy (URS) is almost routinely used in most cases of suspected UTUC. This procedure provides the surgeon with priceless details: endoscopic appearance, selective urine cytology, retrograde imaging, and, most crucially, tissue biopsy. In addition, accurately identifying patients with high-risk tumors would help select those who may benefit from neo-adjuvant chemotherapy (NAC) prior to surgery and others who may be candidates for renal sparing surgery.

This study was aimed at examining the reliability of the various diagnostic parameters in predicting tumor grade in the final pathological report after radical nephroureterectomy.

## PATIENTS AND METHODS

### Study Design and Population

Following the Institutional Research Board approval (RMB-0254-16), data were gathered from computerized medical files using the specific ICD-9 discharge codes, nephrectomy, nephroureterectomy, and ureterectomy.

The charts of all patients with clinically localized UTUC who had undergone RNU at a single tertiary medical center (Rambam Health Care Campus, Haifa, Israel) between January 2000 and March 2020 were reviewed retrospectively. Surgical procedures were performed by an open or minimally invasive approach per the surgeon’s discretion. Patients with no preoperative diagnostic URS, or patients who had received NAC in an intent to avoid potentially confounding impact on pathologic data, were excluded from the study.

### Diagnostic Ureteroscopy

Ipsilateral URS involves four steps in the following order: (1) selective urine cytology collection; (2) retrograde uretero-pyelography; (3) full upper-tract inspection for lesions; and (4) lesion biopsy. A semi-rigid 8/9.8 Fr and 6 Fr flexible ureteroscope (Wolf, Richard Wolf, Berlin, Germany) was used. Tumor biopsies were taken by endoscopic basket, cup-biopsy (BIGopsy Cookmedical®, Bloomington, IN, USA), or forceps.

Biopsies obtained by URS were classified according to the 2004 World Health Organization (WHO) criteria as LG or HG. The biopsy technique and number of biopsies performed were at the treating urologist’s discretion.

### Primary Endpoint

The primary endpoint was the ability to accurately predict tumor grade post-RNU in the final pathological report based on selective cytology, endoscopic appearance, and tissue biopsy grade.

### Secondary Endpoints

Secondary endpoints were as follows:

- To determine the association between demographic data and tumor grade,
- To ascertain the status and disease histology for bladder UC, and
- To determine the association between biopsy grade and final tumor stage.

### Statistical Analysis

The association between URS data and final UTUC pathology results was evaluated using the chi-square test. In calculating sensitivity, specificity, and predictive values, the URS biopsy was used as the diagnostic test, and final pathology in RNU tumor grade (high versus low) was used for disease outcomes. *P*<0.05 was considered statistically significant. All statistical analyses were performed using SPSS software (SPSS 25.0; SPSS Inc. Chicago, IL, USA). The predictive quality of each variable was evaluated according to Equation 1:

## RESULTS

**Figure 1:**
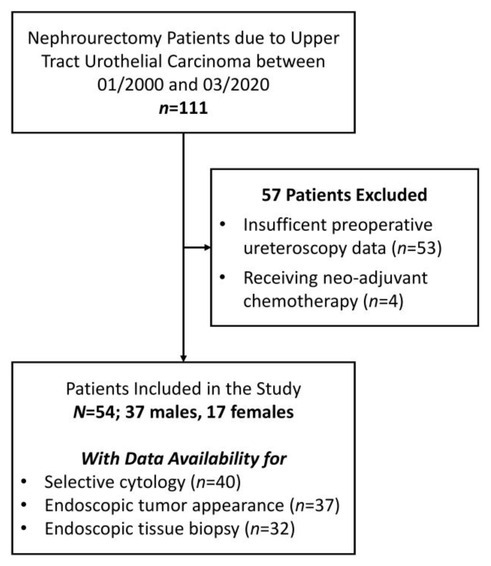
Flow Chart of Patients Who Met the Study Exclusion and Inclusion Criteria.

The single medical center recorded 111 patients undergoing nephroureterectomy due to UTUC between January 2000 and March 2020. Excluded from the study were 57 patients (insufficient preoperative URS data, *n*=53; receiving NAC, *n*=4). The final study population included 54 patients (37 men and 17 women) with a mean age of 71.6±11.21 (range 49–93) years. Selective cytology, endoscopic appearance, and biopsy data were available for 40, 37, and 32 patients, respectively (Figure 1).

Table 1 describes the patients and tumor characteristics. Of note, 10 of the cases were multifocal, and in all of these cases, the renal pelvis was involved. No statistically significant differences were seen between groups regarding age, gender, smoking status, or tumor location (Table 1).

**Table 1:** Demographic Data and Tumor Characteristics on Preoperative Diagnostic Ureteroscopy and Final Pathology.

Parameter	LG in Final Pathology *n* (%)	HG in Final Pathology *n* (%)	*P* Value
Number of cases	23	31	

Age, years	71.9+11.1	71.5+11.3	1
Male	13 (35.1%)	24 (64.9%)	0.118
Female	10 (58.8%)	7 (31.2%)	

Tobacco smoker	17 (73.9%)	13 (41.9%)	0.08

Tumor location			
Ureter	12 (52.2%)	11 (35.4%)	0.16
Renal pelvic	4 (17.4%)	10 (32.2%)	0.34
Renal calyces	3 (13%)	2 (6.4%)	0.64
Multifocal	4 (17.4%)	6 (19.3%)	1
Not documented	0 (0%)	2 (6.4%)	1

Bladder UC	9 (39.1%)	13 (41.9%)	1
Low grade	8 (88.8%)	4 (30.8%)	0.0115
High grade	1 (11.2%)	9 (69.2%)	

Final stage			
T0, Ta, T1	17 (73.9%)	9 (29%) [1 CIS]	0.0021
T2–T4	6 (26.1%)	22 (71%)	

HG, high grade; LG, low grade; SD, standard deviation; UC, urothelial carcinoma.

### Selective Cytology and Final Pathology

The final study cohort comprised 40 patients with selective cytology samples. Of these, 7 (17.5%) with atypical cytology were graded at stage III according to The Paris System (TPS) for reporting urinary cytopathology; final pathology was low grade in 3 and high grade in 4. Due to the weak significance of the atypical cytology, no further analysis was performed for these 7 patients.

Negative urine cytology was noted in 19 patients (47.5%; TPS II and V); final pathology was low grade in 11 (58%) and high grade in 8 (42%). Positive urine cytology was found in 14 patients (35%; TPS IV and VI) with a final pathology of HG in 12 (86%) and LG in 2 (14%).

### Endoscopic Appearance and Final Pathology

Endoscopic tumor appearance was documented in 37 patients. Papillary tumor pattern was noted in 27 (73%), with a final pathology of low grade in 14 (52%) and high grade in 13 (48%). Solid-looking tumor growth was seen in 10 patients (27%), with final pathology of high grade in 4 (40%) and low grade in 6 (60%).


(Equation 1) $$Accuracy=\frac{True\;Positive+True\;Negative}{True\;Positive+True\;Negative+False\;Positive+False\;Negative}$$

### Tissue Biopsy During URS and Final Pathology

An endoscopic biopsy was performed in 32 patients, of which 20 (62.5%) patients were LG. For these 20 patients, final pathology revealed high grade in 8 (40%) and confirmed low grade in 12 (60%). Endoscopic biopsy in the other 12 patients (37.5%) indicated HG tumors, with final pathology revealing 9 (75%) being high grade and 3 (25%) as low grade.

Table 2 provides the sensitivity, specificity, positive predictive value, and negative predictive value for the different parameters.

**Table 2:** Reliability of Diagnostic Ureteroscopy Parameters: Selective Cytology, Lesion Appearance, and Tumor Biopsy.

Parameter (*n*)	Sensitivity (95% CI)	Specificity (95% CI)	PPV (95% CI)	NPV (95% CI)	Test Accuracy (%)
Urine cytology (*n*=33)	0.6 (0.38–0.81)	0.84 (0.65–1.04)	0.86 (0.67–1.04)	0.58 (0.35–0.80)	69.7
Lesion appearance (*n*=37)	0.7 (0.5–0.9)	0.23 (0.03–0.44)	0.4 (0.01–0.7)	0.52 (0.33–0.7)	54
Tumor biopsy (*n*=32)	0.53 (0.29–0.76)	0.8 (0.6–1)	0.75 (0.51–0.99)	0.6 (0.35–0.81)	65

CI, confidence interval; NPV, negative predictive value; PPV, positive predictive value.

### Bladder Urothelial Carcinoma Status and UTUC Grade

Synchronous or metachronous bladder UC was found in 22 (40.7%) patients, and constituted 9/23 (39.1%) of the LG group and 13/31 (41.9%) of the HG group (*P*=1). A statistically significant correlation was observed between bladder UC grade and the final UTUC pathology grade: 8/9 (88%) cases in the LG bladder UC group had low-grade UTUC versus only 4/13 (30.8%) in the high-grade UTUC (*P*=0.011).

### UTUC Grade and Stage Correlation

There was a statistically significant correlation in the entire cohort between high-grade UTUC and invasive disease: 22 (71%) of the patients with high-grade UTUC had T2–T4 stage tumors versus only 9 (29%) in the low-grade UTUC group (*P*=0.0021).

## DISCUSSION

Although RNU is still the gold standard treatment for patients with UTUC, the 2021 European Association of Urology guidelines include conservative management as a treatment option in selected patients (solitary lesions 1 cm, LG, non-muscle-invasive lesions at CT scan, and absence of upper urinary tract dilatation). Renal sparing surgery may be a forced compromise for patients with a solitary kidney, severe chronic kidney disease, or significant comorbidities.^2^

Significant advances in endoscopic technology during the last two decades have contributed to increasing diagnostic accuracy and treatment options in the management of UTUC.^7^ When there is no escape from RNU, peri-adjuvant chemotherapy is a decision of increasing importance. Several retrospective studies evaluated the role of platin-based NAC, and promising pathological downstaging, complete response rates, and mortality reduction have been shown.^6^,^8^–^12^ However, the accuracy of ureteroscopic biopsy and its ability to provide reliable prognostic information is crucial for guiding UTUC treatment.^13^

### Selective Urine Cytology

Since spontaneous bladder cytology has low sensitivity^14^ and high false positive,^15^ selective urine cytology is a common practice in the UTUC diagnostic process. Blute et al.^16^ and Streem et al.^17^ showed a positive brush urine cytology sensitivity of 90% (not determining grade) for the presence of UTUC. Regarding tumor grade, Skolarikos et al. found that positive urine cytology predicts HG in 67% of cases.^18^ Furthermore, in grade 2 (i.e. intermediate) biopsies, the combination of cytology with lesion biopsy improved the sensitivity and specificity of HG tumor detection from 43% to 55% and 23% to 85%, respectively.^18^

We found that positive urine cytology is highly specific in predicting HG tumors (84.6%) but has relatively low sensitivity (60%) in ruling out HG, with a practical accuracy of 69.7%. It is worth noting that our center seeks to avoid achieving endoscopic brush cytology in favor of better visualization later on.

### Lesion Endoscopic Appearance

Papillary endoscopic appearance is estimated to be 85%, while the rest of the lesions are defined as “sessile/solid.”^19^ In our cohort, surgeons described tumor lesions as “papillary” in 73% of the cases. However, only 52% were actually LG. On the other hand, our study provided a slightly better “solid” prediction value, namely 60%. These results amount to a disappointingly low level of accuracy of 54%. El-Hakim et al. revised the urologist impressions of the lesion to predict tumor grade and found a slightly higher accuracy of 70%.^5^ We agree with El-Hakim et al.^5^ that therapeutic decisions should not be based only on tumor appearance, and that a biopsy must be explicitly taken when a solitary lesion is endoscopically treated or NAC is contemplated.

### Tissue Biopsy

Histologic samples obtained by URS provide the best potential for predicting the final pathologic results, with a correlation of 78%–92%.^13^,^20^,^21^ Our study had 20 cases with LG biopsies and 12 HG biopsies (including 3 carcinoma *in situ* [CIS] cases). However, in the final analysis, upgrading and downgrading occurred in 40% and 25% of the LG and HG cases, respectively, providing a relatively lower than expected 65% accuracy. This could be due to several possible reasons. First, upgrading might have occurred in cases where tissue biopsy sampled an LG area in a heterogeneous tumor hiding HG biology. Second, there might have been a non-uniform use of the lesion biopsy device on the part of the user. Lastly, grade diagnosis for biopsy and the final tumor pathology were determined by a different pathologist; UC grade interobserver reproducibility was previously documented as 70%.^22^

With respect to biopsy devices, Lama et al. compared the performance of three contemporary ureteroscopic biopsy devices. His group found that even though a backloaded cup forceps and nitinol basket obtained a higher-quality specimen than standard cup forceps, this did not significantly impact diagnostic accuracy.^23^

### Final Pathological Stage

Previous studies have demonstrated a correlation between UTUC grade and tumor stage. About 68%–100% of UTUC patients with G1 tumors have a tumor stage of ≤pT1, while 62%–100% of patients with G3 tumors have stage ≥pT2.^24^ In our cohort, 74% of LG UC patients had LG tumors in the final pathology, defined as ≤T1, while ~72% of the HG UTUC patients had invasive disease (≥T2). When obtained using a standard endoscopic instrument, URS biopsies rarely contain muscle, which limits stage evaluation accuracy.^7^ As a result, defining the tumor grade provides more relevant pathological information that can aid in predicting tumor stage and can more accurately predict long-term survival at initial biopsy.

There is no doubt regarding the importance of distinguishing between high-risk and low-risk UTUC before the definitive treatment, particularly when considering RNU or NAC. A score combining the different preoperative factors (endoscopic appearance, cytology, biopsy, tumor size, and bladder grade status) can increase prediction accuracy in differentiating high versus low risk (grade and stage) in the final pathology. Due to rarity of UTUC, a multicenter trial is needed.

## LIMITATIONS

Our study had several limitations. First, it was a single-center retrospective cohort study. Second, cytology, endoscopic appearance report, and tissue pathology were not available in all 54 patients, thereby preventing construction of a predictive model using these combined parameters. Third, our results do not include CT urography tumor dimensions, which could have given an additive predictive value for the final pathology. This last-mentioned measurement could not be uniformly defined for several reasons (multifocality, obstructed ureter and lack of secretion phase, the discrepancy between CT findings, and endoscopic description). Fourth, due to the lack of reporting, biopsy instruments could not be included in our data, a known factor in biopsy quality. It is essential to note that upper-tract tissues are routinely re-checked, and when indicated a second biopsy should be obtained. Lastly, patients who had received NAC were excluded from the study due to expected tumor biology changes that might have confounded our results. This could present a challenging problem since NAC is becoming common practice.

## CONCLUSIONS

Direct observation demonstrating papillary lesions, negative cytology, and biopsy with LG findings are of low predictive value for classifying the final pathology UTUC grade and stage. Bladder UC grade, HG biopsy, and positive cytology were found to be better predictors of tumor grade. In light of the rising use of renal sparing surgery for low-risk UTUC and high-risk NAC, a reliable predictive model should be developed that accurately discriminates between high- and low-grade disease.

## Abbreviations

CIS: carcinoma *in situ*
CT: computed tomography
HG: high grade
LG: low grade
NAC: neo-adjuvant chemotherapy
RNU: radical nephroureterectomy
TPS: The Paris System
UC: urothelial carcinoma
URS: ureteroscopy
UTUC: upper-tract urothelial carcinoma
